# Genetic variant of miR-4293 rs12220909 is associated with susceptibility to non-small cell lung cancer in a Chinese Han population

**DOI:** 10.1371/journal.pone.0175666

**Published:** 2017-04-14

**Authors:** Lixia Fan, Linqi Chen, Xiaoling Ni, Shicheng Guo, Yinghui Zhou, Chenji Wang, Yabiao Zheng, Fangyuan Shen, Vijay Kumar Kolluri, Merlin Muktiali, Zhenhong Zhao, Junjie Wu, Dunmei Zhao, Zhenglei He, Xulong Feng, Ziyu Yuan, Juan Zhang, Li Jin, Jiucun Wang, Minghua Wang

**Affiliations:** 1 Department of Biochemistry and Molecular Biology, Medical College, Soochow University, Suzhou, China; 2 MOE Key Laboratory of Contemporary Anthropology and State Key Laboratory of Genetic Engineering, School of Life Sciences, Fudan University, Shanghai, China; 3 Department of General Surgery, Zhongshan Hospital, Fudan University, Shanghai, China; 4 Department of Pneumology, Changhai Hospital of Shanghai, Shanghai, China; 5 Fudan-Taizhou Institute of Health Sciences, Yaocheng Road, Taizhou, Jiangsu, China; Duke Cancer Institute, UNITED STATES

## Abstract

Non-small cell lung cancer is one of the most common cancers and the leading cause of cancer death worldwide. Genetic variants in regulatory regions of some miRNAs might be involved in non-small cell lung cancer susceptibility and survival. rs12220909 (G/C) genetic polymorphism in miR-4293 has been shown to be associated with decreased risk of esophageal squamous cell carcinoma. However, the influence of rs12220909 genetic variation on non-small cell lung cancer susceptibility has not been reported. In order to evaluate the potential association between miR-4293 rs12220909 and non-small cell lung cancer risk in a Chinese population, we performed a case-control study among 998 non-small cell lung cancer cases and 1471 controls. The data shows that miR-4293 rs12220909 was significantly associated with decreased susceptibility to non-small cell lung cancer (GC vs.GG: OR = 0.681, 95%CI = 0.555–0.835, *P* = 2.19E-4; GG vs. GC+CC: OR = 0.687, 95%CI = 0.564–0.837, *P* = 1.95E-4), which indicates that rs12220909 in miR-4293 may play a significant role in the development of non-small cell lung cancer.

## Introduction

Lung cancer is the leading cause of cancer death in both men and women in western society [1]. In recent years, many studies have reported that the incidence and mortality of lung cancer have rapidly increased in developing countries, especially China, where it has become a major public health challenge [2]. Non-small-cell lung cancer (NSCLC) is the most common lung cancer type, which accounts for about 85% lung cancer cases. Although there are many studies describing the development of NSCLC, the mechanism of the disease remains unclear. New evidence points to a strong link between genetic variation, such as SNPs in microRNAs, and NSCLC progression [3–5].

MicroRNAs(miRNAs) are small non-coding RNAs, which are evolutionarily conserved endogenous non-coding RNAs of about 22 nucleotides that regulate gene expression post-transcriptionally through binding to the 3'-untranslated region (3'-UTR) of target mRNAs in a sequence-specific manner, leading to mRNA cleavage [6, 7]. miRNAs are involved in numerous diverse of biologic processes, including cell differentiation, proliferation and apoptosis [8–10]. Previous studies have shown that miRNAs which function as oncogenes or tumor suppressor genes, if aberrantly regulated, might be associated with disease-associated outcomes [11, 12]. Specifically, genetic variants such as SNPs in miRNAs may alter susceptibility to various cancers [13, 14]. A large number of recent studies have revealed that SNPs in miRNA sequences were significantly related to risk of NSCLC [4, 15].

The role of miR-4293 in cancer is not yet known. Gong et al. [16]reported the rs12220909G>C genetic polymorphism is the only SNP locus found in the seed region of miR-4293. According to Gene Ontology and KEGG analysis results, the SNP loci rs12220909G>C not only changes the ability of miR-4293 to combine with target genes, it also alters the quantity and types of target genes miR-4293 acts upon. Additionally, it has been shown that this miR-4293 genetic polymorphism can decrease the risk of esophageal squamous cell carcinoma [17]. Nonetheless, the potential influence of rs12220909 G>C in miR-4293 on the susceptibility to NSCLS has not yet been analyzed. Therefore, we conducted a case-control analysis to explore the potential association between miR-4293 rs12220909G>C and NSCLS risk in a large Chinese Han population.

## Materials and methods

### Study population

In our study, a total of 998 NSCLC patients and 1471 cancer-free controls were enrolled. NSCLC patients were diagnosed, and the disease histologically confirmed by doctors and recruited from two hospitals: Taizhou People’s Hospital and Shanghai Cancer Hospital, without restriction regarding age and gender between January, 2007 and April, 2012. Healthy controls were recruited from Taizhou City over the same period of time. The controls were free from NSCLC and other diseases. All enrolled subjects were unrelated Han ethnic Chinese who had signed a written informed consent. The case-control study is supported by the Institutional Review Board of the School of Life Sciences of Fudan University.

### Candidate SNPs selection and genotyping

On the basis of the public database miRBase (http://www.mirbase.org), we performed a bioinformatics analysis to search SNPs in miRNAs' mature sequences, and the rs12220909 located in miR-4293 came to our attention. It was first reported by Gong et al.[16] that rs12220909 was the only SNP loci located in the miR-4293 seed region. It has been demonstrated that the genetic polymorphism decreased esophageal squamous cell carcinoma risk [17]. However, there were no studies that focused on the association between miR-4293 rs12220909 and NSCLC risk in the Chinese Han population. Thus, the genetic polymorphism in miR-4293 was selected as the candidate variation for the exploration of a connection with NSCLC in this population.

Blood samples were extracted from enrolled subjects and genomic DNA was isolated using the Lai Feng^™^ Genomic DNA blood Miniprep Kit according to the manufacturer’s instructions. A UV spectrophotometer (Nanodrop C723 ND-1000 UV/Vis spectrophotometer) was employed to detect the DNA concentration. Genotyping of miR-4293 rs12220909 genetic polymorphism was completed with MALDI-TOF (Matrix-Assisted Laser Desorption/Ionization-Time of Flight).

The specific primers for PCR and iPLEX reaction were designed by Genotyping Tools & Mass ARRAY Assay Design software. Primers were synthesized by Shanghai Invitrogen Biotechnology. PCR amplification was performed in 384-well plates and shrimp alkaline phosphatase was used to dephosphorylate unincorporated dNTPs in the PCR mixture. After the mass ARRAY iPLEX reaction was performed, iPLEX products with different molecular masses were created by each base of SNP sites. Finally, distinct iPLEX products were identified by matrix-assisted laser desorption/ionization time of flight (MALDI-TOF) and genotyping information of each SNP site was obtained for every subject by MALDI-TOF with TYPER 4.0 software.

### GO and KEGG pathway enrichment analyses

To determine the molecular mechanism behind the decreased susceptibility to NSCLC mediated by miR-4293, in silico analysis was conducted to search for potential loss or gained of genes using the website (http://www.bioguo.org/miRNASNP2/). The ToppGene Suite [18] bioinformatics online tool (available at https://toppgene.cchmc.org/) was subsequently used to identify enriched functional annotation categories for genes lost or gained by the presence of rs12220909 in the miR-4293 seed region. Gene Ontology (GO)[19] terms and Genomes (KEGG) pathway [20]were evaluated. The statistical significance of Gene Ontology (GO) enrichment and pathways analysis were checked by choosing hypergeometric test and Bonferroni—Hochberg false discovery rate (FDR) correction model for multiple test adjustment.

### Statistical analysis

We used chi-square and Student’s t test to evaluate differences in demographic variables between cases and controls. In the analysis procedure, we used the following genetic models: the recessive model (AA+AB vs. BB), the dominant model (AA vs. AB+BB), the allelic model (A vs. B), the homozygous model (AA vs. BB), and the heterozygous model (AA vs. AB); all models assume A is the wild-type allele and B is the mutant allele. The odds ratio (OR) and 95% confidence interval (95%CI) were calculated by logistic regression analysis and were used to estimate the association of SNPs with NSCLC susceptibility. Stratified analyses were conducted by selected demographic variables such as age (< 62, ≥ 62), sex (male, female), smoking status (non-smokers, smokers) and histologic types. *P*-value<0.05 was considered statistically significant. All statistical analysis was performed on SPSS version 18.0 statistical software. A *P*-value < 0.05 was considered statistically significant for the Gene Ontology (GO) enrichment and pathway analysis. The default parameter of the hypergeometric test was selected as the statistical method and Bonferroni—Hochberg was used as the FDR correction method.

## Results

### Characteristics of the study population

A total of 998 NSCLC cases (male, 785; female, 213; mean age, 61.52) and 1471 controls (male, 711; female, 760; mean age, 55.01) were enrolled. The relevant characteristics of subjects were listed in Table 1. Significant differences in terms of gender, age and smoking status were observed between cases and controls (both *P*<0.01) because of the random collection of the community.

**Table 1:** Distribution of characteristics among NSCLC cases and controls.

Variable	Case no. (%) 998	Control no.(%)1471	*P*值^a^
Age (years)	61.52±10.38	55.01±12.52	<0.001^b^
Gender (n)			<0.001
Male	785(78.66)	711 (48.33)	
Female	213(21.34)	760(51.67)	
Smoking status			<0.001
Yes	695 (69.64)	467(31.75)	
No	303 (30.36)	1004 (68.25)	
Pack per year			<0.001
0	303 (30.36)	1004 (68.25)	
≤32	244 (24.45)	413 (28.08)	
>32	449(44.99)	54 (3.67)	
Histology type			
Adenocarcinoma	417 (41.78)		
Squamous cell carcinoma	359 (35.97)		
Other non-small cell lung cancer^c^	56 (5.61)		
Small cell cancer	67 (6.71)		

^**a**^ Use double side chi-square test

^**b**^ Use independent sample t-test

^**c**^ Including large cell carcinoma, alveolar carcinoma and undifferentiated carcinoma

### Association analysis of miR-4293 rs12220909 with NSCLC susceptibility

The genotype, allele frequencies distributions and additive model of rs12220909 are showed in Table 2. The rs12220909 was in Hardy-Weinberg equilibrium (*P*>0.05, data not shown). Genotype and allele distributions of miR-4293 rs12220909 polymorphism showed significant difference between cases and controls. The GC genotype is associated with a decrease NSCLC susceptibility (OR = 0.681, 95%CI = 0.555–0.835, *P* = 2.19×10^−4^). The frequency of the C allele was significantly higher than the G allele in the cancer patients vs the controls (OR = 0.734, 95%CI = 0.616–0.874, *P* = 0.04). A protective role in NSCLC was found for the miR-4293 rs12220909 heterozygous mutation under the dominant model (OR = 0.687, 95%CI = 0.564–0.837, *P* = 1.95×10^−4^) and the additive model (OR = 0.752, 95%CI = 0.608–0.931, P = 5.9 ×10^−4^).

**Table 2:** Genotype frequencies of miR4293 rs12220909 and association with NSCLC.

Genotypes	Controls no. (%)	Cases no. (%)	OR (95% CI) ^a^	*P*-value^b^
miR-4293 rs12220909	N = 1454	N = 995		
GG	999(68.7)	753 (75.7)	1.00	
GC	419 (28.8)	220 (22.1)	0.681 (0.555–0.835)	2.19×10^−4^
CC	36 (2.5)	22 (2.2)	0.762 (0.426–1.366)	0.362
Dominant model (GG vs. GC+CC)	999/455	753/242	0.687 (0.564–0.837)	1.95×10^−4^
Recessive model (GG+GC vs.CC)	1418/36	973/22	0.843 (0.472–1.505)	0.563
G	2417 (83.1)	1726 (86.7)	1.00	
C	491 (16.9)	264 (13.3)	0.734(0.616–0.874)	0.001
Additive model (GG/GC/CC)	999/419/36	753/220/22	0.752(0.608–0.931)	5.9×10^−4^

^a^ OR: Odds ratio; CI: Confidence interval

^b^ adjustment for age, gender, smoking status using the two categories of logistic regression analysis

### Stratified analysis of miR-4293 rs12220909 with NSCLC risk

We further performed stratification analysis with selected demographic variables. The characteristics of gender, age, smoking status and histologic types were included in the following stratified analyses (Tables 3 and 4). For miR-4293 rs12220909G>C, GC genotypes were observed with obvious decreasing NSCLC susceptibility among male (OR = 0.620, 95%CI = 0.484–0.793, *P* = 1.46×10^−4^), lower age (OR = 0.743, 95%CI = 0.563–0.982, *P* = 0.037), higher age (OR = 0.635, 95%CI = 0.470–0.858, *P* = 0.003), non- smoker (OR = 0.700, 95%CI = 0.513–0.954, *P* = 0.024), smoker (OR = 0.659, 95%CI = 0.503–0.863, *P* = 0.002) and squamous cell carcinoma group (OR = 0.561, 95%CI = 0.409–0.769, *P* = 3.32×10^−4^), adenocarcinoma group (OR = 0.749, 95%CI = 0.579–0.971, *P* = 0.029). The results indicated GC/CC genotypes only have a significantly different influence on male and female groups.

**Table 3:** Stratified results for pri-miR-4293 rs12220909 G>C in the heterozygous model.

Variable	GG/GC		OR ^a^(95% CI)	*P* ^b^
	Case	Control		
Age				
< 62	368/111	640/265	0.743 (0.563–0.982)	0.037*
≥62	385/109	359/154	0.635 (0.470–0.858)	0.003*
Gender (n)				
Male	596/168	481/203	0.620 (0.484–0.793)	1.46×10^−4^*
Female	157/52	518/216	0.814 (0.572–1.158)	0.252
Smoking				
Yes	522/155	311/138	0.659 (0.503–0.863)	0.002*
No	231/65	688/281	0.700 (0.513–0.954)	0.024*
Squamous cell carcinoma	281/72	999/419	0.561 (0.409–0.769)	3.32×10^−4^*
Adenocarcinoma	306/98	999/419	0.749 (0.579–0.971)	0.029*

^a^ OR: Odds ratio; CI: Confidence interval

^b^ adjustment for age, gender, smoking status using the two categories of logistic regression analysis

**Table 4:** Stratified results for pri-miR-4293 rs12220909 G>C in the dominant model.

Variable	GG/GC+CC		OR ^a^(95% CI)	*P* ^b^
	Case	Control		
Age				
<62	368/124	640/289	0.687 (0.579–0.989)	0.041*
≥62	385/118	359/166	0.633 (0.473–0.849)	0.002*
Gender(n)				
Male	596/187	481/220	0.636 (0.501–0.807)	1.98×10^−4^*
Female	157/55	518/235	0.793 (0.561–1.119)	0.187
Smoking				
Yes	522/172	311/150	0.672 (0.517–0.873)	0.003*
No	231/70	688/305	0.696 (0.515–0.940)	0.018*
Squamous cell carcinoma	281/78	999/455	0.561 (0.413–0.762)	2.17×10^−4^*
Adenocarcinoma	306/108	999/455	0.759 (0.591–0.935)	0.031*

^a^ OR: Odds ratio; CI: Confidence interval

^b^ adjustment for age, gender, smoking status using the two categories of logistic regression analysis

### Functional enrichment and signaling pathway analyses

To further determine the molecular mechanism behind miR-4293-mediated increased risk of NSCLC in our population, in silico studies were performed to search for target genes that were lost and gained. The miR-4293 gained 288 and lost 1875 genes due to rs12220909 G>C. We then used the Toppgene online tool for GO enrichment and pathway analysis, which showed that the genes regulated by miR-4293 rs12220909 were significantly enriched in amino acid transmembrane transporter activity and neutral amino acid transmembrane transporter activity (S1 and S2 Tables).

## Discussion

In the present study, we explored the potential association of the miR-4293 rs12220909 genetic polymorphism with NSCLC risk in a Chinese population. We found that the miR-4293 rs12220909 is significantly associated with decreased susceptibility to NSCLC.

Gong et al.[16] have reported that the rs12220909 polymorphism in miR-4293 affected the binding activity of miR-4293 to target genes. Additionally, it has been determined that this genetic polymorphism significantly decreases risk of esophageal squamous cell carcinoma (OR = 0.77, 95%CI = 0.61–0.97) [17]. However, the potential influence of the rs12220909 G>C genetic polymorphism in miR-4293 on NSCLC susceptibility has not been analyzed. The study found that the GC/CC genotypes of miR-4293 rs12220909 were associated significantly with decreased NSCLC susceptibility (OR = 0.681, 95%CI = 0.555–0.835, *P* = 2.19×10^−4^), which indicates that the mutant allele C of rs12220909 can influence the function of miR-4293 (OR = 0.734, 95%CI = 0.616–0.874, *P* = 0.001). Similar results were also observed in the follow-up stratified analysis. However, we did not observe a significant association of miR-4293 rs12220909 with NSCLC risk among the female population in our study. This result indicates that SNP rs12220909 makes NSCLC a different susceptibility in gender population.

In silico studies showed that miR-4293 would potentially lose 1, 875 target genes and gain only 288 target genes after the G was replaced by the C allele in rs12220909. These results suggest that a SNP located in the miRNA seed region may change the quantity of target genes, which would lead to miRNA functional change. Based on our results, we speculate that the mutant allele C could increase the binding between miR-4293 and some cancer-related oncogenes, and may reveal the functional mechanism of the protective role of miR-4293 rs12220909G>C in NSCLC development. In addition, GO enrichment and pathway analysis results suggest that miR-4293 rs12220909-mediated genes are significantly enriched in the GO category; these genes are involved in molecular functions such as amino acid transmembrane transporter activity and neutral amino acid transmembrane transporter activity. SLC43A2 and SLC7A5, members of the L-type amino acid transporter (LAT) family, are responsible for the majority of cellular leucine uptake. It has been reported that SLC43A2 and SLC7A5 show increased expression in many types of cancer, and play a critical role in controlling protein translation and cell growth through the mTORC1 pathway [21]. We speculate that such gain-of-function of these genes via miR-4293 rs12220909 is associated with decreased NSCLC susceptibility. In order to verify our hypothesis, we plan to focus on target candidates likely to be related to NSCLC to validate the functional mechanism.

In conclusion, our results provide the first evidence that rs12220909G>C polymorphism in the miR-4293 seed region is associated with decreased NSCLC risk in a Chinese population, and may play significant role in the process of NSCLC.

## Supporting information

S1 Table Functional enrichment and signaling pathway analyses of gained genes. (XLSX)

S2 Table Functional enrichment and signaling pathway analyses of lost genes. (XLSX)
